# Parental Leave Policies in the Top 20 US Hospitals: A Call for Inclusivity and Improvement

**DOI:** 10.1089/whr.2023.0010

**Published:** 2023-04-17

**Authors:** Molly B. Kraus, Aqsa Khan, Natalie Strand, Shivani G. Mukkamala, Kaley B. McMullen, Camara M. Sharperson, Monica W. Harbell

**Affiliations:** ^1^ Department of Anesthesiology and Perioperative Medicine, Mayo Clinic, Phoenix, Arizona, USA.; ^2^ Department of Urology, Mayo Clinic, Phoenix, Arizona, USA.; ^3^ Department of Anesthesiology, Children's Healthcare of Atlanta and Emory University, Atlanta, Georgia, USA.; ^4^ Mayo Clinic Alix School of Medicine, Phoenix, Arizona, USA.; ^5^ Emory School of Medicine, Atlanta, Georgia, USA.

**Keywords:** parental leave, diversity and inclusion, academic medicine

## Abstract

**Objective:**

To evaluate and compare parental leave policies from the top United States (US) hospitals with a focus on inclusivity of all types of parents.

**Methods:**

In September and October of 2021, the parental leave policies of the top 20 US hospitals, ranked by the 2021 US News & World report, were evaluated. Parental leave policies were obtained and reviewed through the hospitals' public websites. Hospitals' Human Relations (HR) departments were contacted to confirm the policies. Hospital policies were scored against a rubric created by the authors.

**Results:**

Among the top US hospitals (21 total hospitals), 17 (81%) had publicly available policies, and one policy was obtained by contacting HR. Fourteen of the 18 hospitals (77.8%) had a parental leave policy distinctive from short-term disability and offered paid paternity or partner leave. Thirteen hospitals (72.2%) offered parental leave for parents whose children were carried through surrogacy. Fourteen hospitals (77.8%) included adoptive parents; however, only five hospitals (27.8%) specifically included foster parents. The average paid leave for birthing mothers was 7.9 weeks compared to 6.6 weeks for nonbirthing parents. Only three hospitals offered the same leave for birthing and nonbirthing parents.

**Conclusion:**

While a few of the top 20 hospitals have paid parental leave policies that are inclusive and equivalent to all parents, many do not and represent an area for improvement. As healthcare industry leaders, these hospitals should strive for inclusive parental leave policies that care for their employees with the same high standards they set for caring for patients.

## Introduction

Parental leave has shown to benefit the health and well-being of parents and children.^1–6^ The American Academy of Pediatrics (AAP) serves as an advocate for parents and children and endorses 12 weeks of paid parental leave, as this time is essential to children's health.^7^ Studies have shown paid parental leave of increased duration correlates with multiple benefits, including successful breastfeeding initiation and duration, reductions in maternal postpartum depression, and improved marriage quality postchildbirth.^6^

The United States (US) is the only industrialized nation that does not have a universal policy of at least 12 weeks of paid maternity leave.^8^ The Family and Medical Leave Act of 1993 provides up to 12 weeks of job protection for serious illness in the family, perinatal care, childbirth, newborn care, or the care of a newly adopted child; however, this does not require that the leave is paid.^9^ There has been movement toward establishing paid leave policies; currently, six states (California, New Jersey, Rhode Island, New York, Washington, and Massachusetts) and the District of Columbia have instituted paid parental leave policies and three states have plans to do so before 2024 (Connecticut, Oregon, Colorado).^10^ The technology industry has recognized the benefits of parental leave for all parents and have instituted generous and inclusive policies that apply to both parents and include paid leave for 12 weeks or more.^11^

The healthcare field, however, has lagged behind in providing generous and inclusive parental leave. Previous studies have highlighted the need to improve parental leave policies within medical schools, residency programs, and the top hospitals and cancer centers.^12–14^ This study seeks to collectively summarize, evaluate, and compare the parental leave policies for physicians among the top US hospitals with a focus on the inclusivity of all ways individuals become parents, regardless of whether or not they are the birthing mother, father, same-sex partner, or have a child through surrogacy, foster care, or adoption.

## Methods

In September and October of 2021, we evaluated the parental leave policies for physicians of the top 20 hospitals in the United States, as identified by the 2021–2022 US News & World Report (USNWR).^15^ We chose to use the USNWR ranking as a source to identify the top 20 hospitals as USNWR rank hospitals in the United States based on multiple dimensions using data obtained from the American Hospital Association Annual Survey, National Cancer Institute's list of NIH-designated centers, American Nurses Credentialing Center's list of Nurse Magnet hospitals, Doximity, Medicare claims dataset, and Hospital Consumer Assessment of Healthcare Providers and Systems (HCAHPS).^16^ While other ranking systems exist, the hospitals identified by USNWR are considered to be leaders in medicine.

Since the seventh hospital was New York Presbyterian-Columbia-Cornell, we included both Cornell and Columbia's policies for physicians in the results and analysis for a total of 21 hospitals. The leave policy for employed physicians was obtained through the internet by searching the hospitals' public website and searching relevant terms such as “parental leave policy,” “family leave policy,” “maternity leave policy,” and “faculty and staff physicians.” Each policy was reviewed by two separate investigators to ensure agreement in interpretation of the policy. If any detail of the policy was unclear or if there was disagreement, it was reviewed by two additional investigators and discussed until agreement was found. When only a faculty policy was available, it was included and noted in the results.

To the best of our knowledge, we obtained the most current policy available at the time. A list of publicly available policies is available as Supplementary Table S1. The Human Relations (HR) departments at all the hospitals were contacted to confirm details of the policies. When the policy was not available publicly online, we asked the HR departments to provide the policy and confirm the details. Multiple attempts were made to contact HR departments.

The 2020 healthcare equality index (HEI), which evaluates a healthcare facilities' policies and practices related to equity and inclusion of LGBTQ (lesbian, gay, bisexual, transgender, queer) patients and employees, was also obtained for each hospital.^17^ The top score achievable on the HEI is 100; and scores between 80 and 95 points are considered “top performers.”

We reviewed each policy to identify if there was a separate parental leave policy from short-term disability (STD) and if STD is the only leave offered for birthing mothers. Details of the policies were extracted, such as length of leave, paid versus unpaid time, and availability to nonbirthing parents such as partners, adoptive or foster parents, and parents who use a surrogate. When policies used terms like “parent” to give time to “bond with new child,” we assumed this would be inclusive of parents who have children through surrogacy. Policies were deemed inclusive of adoptive or foster parents only if the term “adoption” or “foster care” was used in the policy.

When policies used the term “birthing mother,” we assumed that it would not be inclusive of parents who have children through surrogacy or adoption. Eligibility requirements to take leave were also noted. We evaluated if legislation regarding parental leave existed in the states or cities where the hospitals are located. When calculating the average length of paid leave for the birthing parent and nonbirthing parent, if the policy specified different leave amounts for primary and secondary parents, the amount of leave for the primary parent was used for the birthing parent, while the amount for the secondary parent was used for the nonbirthing parent.

Descriptive analyses were conducted to compare characteristics of parental leave policies. Frequencies and proportions were used to summarize variables in the scoring rubric. Mean and standard deviation were reported for total scores. Comparisons of policies between hospitals with and without state policies were made using *t* test. Statistical significance was defined as *α* = 0.05. All analyses were carried out using SAS 9.4 (Cary, NC).

## Results

Of the 21 hospitals, 17 hospitals (81%) had publicly available policies that could be obtained from the internet (Supplementary Table S1). One hospital (Rush) was not available publicly, but was provided by the HR department. A total of 18 policies were obtained.

HR at 10 hospitals confirmed the details of the policies. Two hospitals would not confirm the policy details to nonemployees. The remaining HR departments could not be reached to confirm details, despite multiple attempts. At three of the hospitals, there was no available information on parental leave or STD through the internet or by contacting the HR departments.

Fourteen of the 18 hospitals (77.8%) had a parental leave policy distinctive from STD. Of these hospitals, the average paid leave for the birthing parent was 7.9 weeks. Three of these hospitals (16.7%) indicated that they only provide a partial amount of the individual's salary. Eleven (52.3%) of the hospitals were in locations that had state or local legislation requiring paid parental leave, including New York, Massachusetts, California, and the District of Columbia.^10^

Of the 10 hospitals in locations that do not have legislation dictating parental leave, six hospitals (60%) had parental leave policies separate from STD. Fourteen of the 18 hospitals (77.8%) offered paid paternity leave or partner leave, with an average length of 6.6 weeks. Only three hospitals (17%) offered the father/partner the same parental leave as the birthing parent. Fourteen policies (77.8%) mentioned the inclusion of adoptive parents. Five hospital policies (27.8%) specifically included foster parents. Thirteen hospitals (72.2%) offered parental leave for parents whose children were carried through surrogacy. The 2020 HEI score was available for 19 of the 21 hospitals and ranged from 90 to 100 with an average score of 98.9. Table 1 summarizes policy details gathered from these 21 hospitals.

**Table 1.** Details of Parental Leave Policy by Top 20 Hospitals as Ranked by United States News and World Report

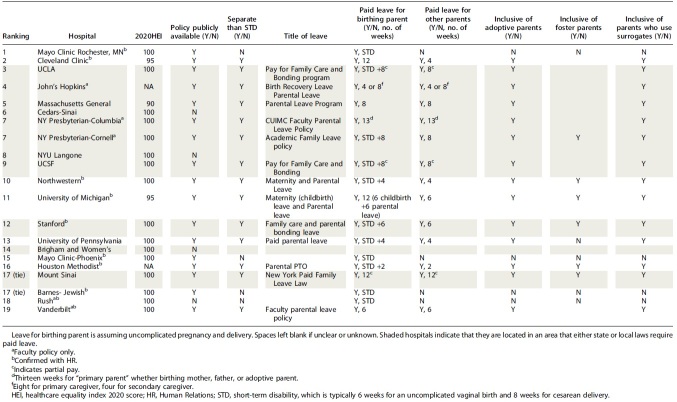

## Discussion

Parental leave is well known to have benefits for both parents and child.^6,18–22^ For decades, paid maternity leave of 12 weeks or more has been established in every industrialized nation, except the United States.^6^ In our study, the average leave for birthing parents in our study was 7.9 weeks. This falls short of the 12 weeks recommended by the AAP.^7^ Paid parental leave has proven to increase women's retention in the workforce.^1,3^ We believe that an increase in the length of paid parental leave to 12 weeks may improve retention of women in medicine.

Within 6 years of completing graduate medical training, 30.6% of women physicians with children work part time compared to 4.6% of men physicians with children, and a significant number of women leave medicine altogether within this time frame.^4^ Houser reported that those who take paid parental leave are 93% more likely to be working 9–12 months after childbirth than those who do not take any leave at all.^1^ Furthermore, another study reported that increasing the duration of paid leave increases the number of hours worked and wages earned in the following 1–3 years after childbirth.^5^

Studies have also shown increased benefit when nonbirthing parents, such as fathers or partners, are encouraged to take leave. Fathers who take time off following the birth of a child, especially 2 weeks or more, are more likely to be involved in the activities of childcare.^2^ Although 14 of the top hospitals offered some paid leave for nonbirthing parents, there was high variability in the duration of paid leave, ranging from 2 to 13 weeks.

Inclusive parental leave policies that are equal for both parents also promote gender equity. In our study, only three hospitals offered the same leave to all parents regardless of birthing or “primary” parent status. Policies that are inclusive of all parents allow the nonbirthing parent to have a greater child-rearing role. This can facilitate the mothers' return and participation in the workforce, helping promote gender equity.^23^ If it becomes the norm for all parents to take leave after the birth of a child, we speculate that there will likely be less bias against women physicians who take leave.^24^

It has been shown that women physicians spend more time on domestic activities and are more likely than their male counterparts to take time off if there is a disruption in the family's daily childcare.^25^ It has also been demonstrated that these women do not achieve the same career success as their male counterparts.^25^ More inclusive parental leave policies may be the first step in providing a more level playing field in women's professional careers. When parental leave policies distinguish birthing and nonbirthing parents or primary and secondary parent, they are promoting long held gender stereotypes.

Although most of these top hospitals had parental leave policies distinctive from short-term disability, few were equal for all types of parents. Given that 25% of female physicians will have infertility, which is markedly higher than the general US population, we assert that inclusive policies that include adoptive parents and those using surrogates may be even more important in physicians.^22,26,27^

Institutions that provide leave only based on short-term disability, which is the case at 4 (22%) of these top hospitals, need to create separate parental leave policies that are inclusive to all parents. Given that the HEI is a measure of equity and inclusivity of LGBTQ patients and employees and given the high HEI scores for these top hospitals, we expected these hospitals to have inclusive parental leave policies to adequately support this employee population such as inclusion of adoptive parents or parents using surrogacy. This was not the case with all the top 20 hospitals.

A change in culture and policies surrounding parental leave in healthcare in the United States is needed now more than ever.^28^ The COVID-19 pandemic has reversed women's progress toward equity in the workforce. Women have been more than four times more likely to leave the workforce than men, mostly due to childcare needs.^29^ A group of women physicians and scientists recently warned that the pandemic could lead to “hemorrhaging” of women from academia.^30^

Changes to policies to improve retention of women physicians should be a top priority for healthcare organizations right now. The technology industry has been a pioneer in improving parental leave policies. Companies such as Netflix, Microsoft, Adobe, Google, and Facebook provide paid parental leave ranging from 12 weeks to a full year. Their policies are generous and inclusive and open to nonbirthing parents, such as fathers and new parents through surrogacy, adoption, or foster care.^31–34^ Hospitals should look to these technology companies when designing their parental leave policies.

In summary, we suggest that the ideal parental leave policy be publically available and easily accessable on the internet. The ideal policy would be paid and separate from short-term diabaility. It would use the term parent and would be available to both parents if both are employed by the same hospital. An ideal policy would not distinguish between birthing versus nonbirthing parent, mothers versus fathers, and primary versus secondary caregiviers, and it would not define if a child is biological. Although implementing more inclusive parental leave policies may impact staffing, particularly in small departments, we believe these challenges are outweighed by the benefits that can be gained in faculty recruitment, satisfaction, and retention.

This study was limited in that we only analyzed the top 20 hospitals in the United States as defined by USNWR. While USNWR is one method of evaluating hospitals, there are multiple different metrics and ways to measure “top” hospitals and USNWR may not be an exhaustive list. Although parental leave policies at other US hospitals may differ from the policies included in this study, we chose to look at these top hospitals as they are considered leaders in medicine. Given that our leading healthcare institutions have room for improvement to provide optimal parental leave for our workforce, it may suggest that other hospitals likely do as well.

Another limitation of this study is that only available policies were analyzed. It is possible that individual department policies may be more inclusive. Hospitals' parental leave policies were analyzed in 2021, and this study does not take into account recent changes hospitals may have made in leave policies since our analysis. Furthermore, faculty may also have appointments at several institutions (*e.g.,* the hospital, academic institutions, Veterans Affairs) with differing parental leave benefits or may be part of a multispecialty faculty practice that is completely separate from the hospital, which were not able to be captured in this study.

In addition, many of these hospitals are affiliated with academic institutions. If a physician has a position at the medical school, they may have a different policy than offered to employed physicians at the hospital. It would be beneficial to have a validated grading system to measure and compare the equitability and inclusivity of parental leave policies among different institutions or to compare policies to a “gold standard”; however, none exists at this time. Despite these limitations, we view this study as a first step in understanding parental leave policies for physicians and believe that the most inclusive parental leave should be available at the institutional level.

Although all human resource departments were contacted to verify the policy, some could not be reached or would not verify the policy. We believe that parental leave policies should be publicly available, so applicants can access them anonymously and easily. Ninety-two percent of respondents in a survey of medical students reported wanting parental leave policies to be formally presented in interviews and 68% reported feeling uncomfortable asking about parental leave.^35^ Similarly, qualified faculty candidates could also feel uncomfortable asking about parental leave policies during the interview process, although it could be important to them when choosing a position.

## Conclusion

Many hospitals have parental leave policies that are not equal for or inclusive of all types of parents. Changes are needed to recruit and retain as many parents in the workforce as possible. While this was not an exhaustive list, these top ranked hospitals have national influence and financial resources, and excel in healthcare. As leaders in the healthcare industry, these hospitals should strive for inclusive parental leave policies that care for their employees with the same high standards they set for caring for patients.

## Authors' Contributions

M.B.K.: study conception and design, data curation and analysis, article writing, and final approval of the article. A.K.: article writing and final approval of the article. N.S.: article writing and final approval of the article. S.G.M.: data curation and analysis, article writing, and final approval of the article. K.B.M.: data curation and analysis, article writing, and final approval of the article. C.M.S.: data curation and analysis, article writing, and final approval of the article. M.W.H.: article writing and final approval of the article.

## Consent

Permission was obtained from USNWR to use their hospital rankings for this article.

## Supplementary Material

Supplemental data

## Abbreviations Used

AAP: American Academy of Pediatrics
HCAHPS: Hospital Consumer Assessment of Healthcare Providers and Systems
HEI: healthcare equality index
HR: Human Resources
LGBTQ: lesbian, gay, bisexual, transgender, queer
STD: short-term disability
US: United States
USNWR: United States News & World Report
