# Supplementary material for: Parental Leave Policies in the Top 20 US Hospitals: A Call for Inclusivity and Improvement
Source: Womens Health Rep (New Rochelle). 2023 Apr 17;4(1):162–8. doi: 10.1089/whr.2023.0010 (PMC10122222; doi:10.1089/whr.2023.0010)
Supplement: Supplemental data [file Suppl_TableS1.docx]

**Supplemental Digital Content 1.**  Publicly available paid parental leave policies of the top 20 US hospitals

| **Rank** | **Hospital name** | **Parental leave policy link** |
| --- | --- | --- |
| **1 & 15** | Mayo Clinic | Mayo Clinic. Summary Plan Description: Mayo Clinic Health & Welfare Benefits Plan. <https://mcforms.mayo.edu/mc5500-mc5599/mc5500-117.pdf>. Published 2021. Accessed October 13, 2021. |
| **2** | Cleveland Clinic | Caring for Caregivers, Their Families and Their Newborns with Paid Leave for New Parents [press release]. Cleveland Clinic 2019. |
| **3 & 9** | University of California, Los Angeles and San Francisco | Regents of the University of California. Pay for Family Care and Bonding. <https://ucnet.universityofcalifornia.edu/compensation-and-benefits/other-benefits/pay-for-family-care-bonding.html>. Published 2021. Accessed October 4, 2021.  University of California. Pregnancy, Newborn Child and Adopted Child. <https://ucnet.universityofcalifornia.edu/forms/pdf/pregnancy-newborn-child-and-adopted-child.pdf>. Published 2021. Accessed October 4, 2021. |
| **4** | Johns Hopkins | John Hopkins School of Medicine. Faculty Parental Leave Guidelines. <https://www.hopkinsmedicine.org/som/faculty/policies/facultypolicies/parental_leave.html>. Published 2021. Accessed October 4, 2021.  John Hopkins Medicine. Nonrepresented Employees of the John Hopkins Hospital and the Johns Hopkins Health System Corporation. <https://www.hopkinsmedicine.org/human_resources/_docs/benefits/2021-jhh-jhhsc-nonunion-benefits-guide.pdf.> Published 2021. Accessed October 4, 2021. |
| **5** | Massachusetts General | Massachusetts General Hospital. Parental Leave Program. <https://www.massgeneral.org/faculty-development/work-life/parental-leave-program>. Published 2021. Accessed October 4, 2021. |
| **6** | Cedars-Sinai | N/A |
| **7** | New York Presbyterian   - Columbia - Cornell | Columbia University Irving Medical Center. CUIMC Faculty Parental Leave Policy. <https://www.vagelos.columbia.edu/about-us/explore-vp-s/leadership-and-administration/academic-affairs/policies-tools-and-resources/cuimc-faculty-parental-leave-policy>. Published 2021. Accessed October 12, 2021  Weill Cornell Medicine. Family and Medical Leave, Salary Continuance and Academic Family Leave Policies. <https://faculty.weill.cornell.edu/family-and-medical-leave-salary-continuance-and-academic-family-leave-policies>. Published 2019. Accessed October 12, 2021. |
| **8** | NYU Langone | N/A |
| **10** | Northwestern | Northwestern Medicine. Benefits. <https://jobs.nm.org/benefits>. Published 2021. Accessed October 8, 2021. |
| **11** | University of Michigan | University of Michigan Human Resources. Maternity (Childbirth) and Parental Leave. <https://hr.umich.edu/working-u-m/my-employment/leaves-absence/maternity-childbirth-parental-leave>. Published 2021. Accessed October 5, 2021. |
| **12** | Stanford | Stanford University. Family Care & Parental Bonding Leave. <https://cardinalatwork.stanford.edu/benefits-rewards/time-away/leaves-of-absence/family-care-leave>. Published 2021. Accessed October 5, 2021. |
| **13** | University of Pennsylvania | The University of Pennsylvania. Paid Parental Leave. <https://www.hr.upenn.edu/policies-and-procedures/policy-manual/time-off/paid-parental-leave-policy>. Published 2021. Accessed October 5, 2021. |
| **14** | Brigham and Women’s | N/A |
| **16** | Houston Methodist | Houston Methodist. Parental PTO. <https://hrportal.ehr.com/houstonmethodist/Home/Time-Off-Career/Time-Off/Parental-PTO>. Published 2020. Accessed October 13, 2021. |
| **17** | Mount Sinai | The Mount Sinai Health System. Federal Family and Medical Leaves of Absence and New York Paid Family Leave. <https://icahn.mssm.edu/files/ISMMS/Assets/About%20the%20School/Ombuds%20Office/HRpolicy-leaves-absence-FMLA-PFL.pdf>. Published 2018. Accessed October 13, 2021. |
| **18** | Barnes-Jewish | BJC Healthcare. Disability Insurance. <https://www.bjctotalrewards.org/Benefits/Disability-Insurance>. Accessed October 13, 2021.  BJC Healthcare. Leave of Absence. <https://www.bjctotalrewards.org/Work-Life/Leave-of-Absence>. Accessed October 13, 2021. |
| **19** | Rush | N/A |
| **20** | Vanderbilt | Vanderbilt Unviersity Medical Center. Faculty Parental Leave Policy. <https://www.vumc.org/faculty/faculty-parental-leave-policy>. Published 2021. Accessed October 3, 2021. |
